# Total Antioxidant Capacity from Dietary Supplement Decreases the Likelihood of Having Metabolic Syndrome in Korean Adults

**DOI:** 10.3390/nu9101055

**Published:** 2017-09-22

**Authors:** Subeen Kim, YoonJu Song, Jung Eun Lee, Shinyoung Jun, Sangah Shin, Gyung-Ah Wie, Yoon Hee Cho, Hyojee Joung

**Affiliations:** 1 Department of Public Health, Graduate School of Public Health, Seoul National University, Seoul 08826, Korea; subeen2466@snu.ac.kr; 2 Major of Food and Nutrition, School of Human Ecology, The Catholic University of Korea, Bucheon 14662, Korea; yjsong@catholic.ac.kr; 3 Department of Food and Nutrition, College of Human Ecology, Seoul National University, Seoul 08826, Korea; jungelee@snu.ac.kr; 4 Department of Nutrition Science, Purdue University, West Lafayette, IN 47907, USA; jun24@purdue.edu; 5 Department of Food and Nutrition, Chung-Ang University, Gyeonggi-do 17546, Korea; ivory8320@cau.ac.kr; 6 Department of Clinical Nutrition, Research Institute & Hospital, National Cancer Center, Goyang-si 10408, Korea; gawie@ncc.re.kr; 7 Department of Biomedical and Pharmaceutical Sciences, The University of Montana, Missoula, MT 59812, USA; yoonhee.cho@umontana.edu; 8 Institute of Health and Environment, Seoul National University, Seoul 08826, Korea

**Keywords:** dietary supplements, antioxidant vitamins, total antioxidant capacity, metabolic syndrome, Korean adults

## Abstract

This study was conducted to estimate antioxidant vitamin intake and total antioxidant capacity (TAC) from diet and dietary supplements and to examine their association with metabolic syndrome (MetS) in Korean adults. Out of 6308 adults 19~64 years old from the 2010~2011 Korea National Health and Nutrition Examination Survey, 1847 adults were classified as dietary supplement users and the other 4461 adults were classified as non-users. Antioxidant intake and TAC from diet and dietary supplements were estimated using dietary intake data and linked with the antioxidant and TAC database for common Korean foods. The prevalence of MetS was lower in dietary supplement users (odds ratio (OR) = 0.82; 95% confidence interval (CI), 0.68–0.98) than that in non-users. Among dietary supplement users, a lower prevalence of MetS was observed in the highest tertile for vitamin A (OR = 0.72; 95% CI, 0.53–0.99) and vitamin E (OR = 0.74; 95% CI, 0.55–0.99) intake than that in the lowest tertile among non-users. Subjects in the highest tertile of TAC among dietary supplement users showed a lower prevalence of MetS (OR = 0.72; 95% CI, 0.52–0.99) than non-users. The results imply that intake of vitamin A, vitamin E, and TAC from dietary supplements might have a protective effect on MetS among Korean adults.

## 1. Introduction

Metabolic syndrome (MetS) is a cluster of risk factors for cardiovascular diseases and diabetes, such as elevated blood pressure, hyperglycemia, high triglyceride levels, low high-density lipoprotein (HDL) cholesterol levels, and abdominal obesity [1]. MetS is diagnosed when three or more of the above risk factors are present. The prevalence of MetS steadily increased in Korea from 24.9% in 1998 to 31.3% in 2007, and it was maintained as high as 28.9% in 2013, according to the Korean Centers for Disease Control and Prevention [2,3]. Among contributing risk factors for MetS, including heredity, aging, diet, physical inactivity, irregular sleep, and excessive alcohol consumption [4,5], dietary factors are known to play an important role in the development and prevention of MetS [6].

Antioxidants, or substances that prevent oxidation of other molecules [7], are considered to be dietary factors associated with MetS. Antioxidants include phytochemicals, vitamins (A, C, E), and selenium, which are mainly found in plant foods such as fruits, vegetables, and tea [8]. Antioxidant intake has been reported to reduce oxidative stress and alleviate the inflammatory response, which are involved in the development of MetS [7,9].

Antioxidants are consumed during meals and via dietary supplements. According to the 2015 Korea National Health and Nutrition Examination Survey (KNHANES), 35.5% of males and 46.2% of females consumed antioxidants from dietary supplements [10]. Notably, vitamin and mineral supplements containing antioxidants were the most commonly consumed dietary supplements among Koreans [11]. In the United States, where more than 50% of people have reportedly consumed dietary supplements, 54% of the vitamin C intake and 64% of the vitamin E intake were reported to be supplied by dietary supplements, indicating that dietary supplements are significant contributors to total antioxidant intake [12]. However, the characteristics of antioxidant intake from dietary supplements have not been examined yet in Korea, despite the high consumption of vitamin and mineral supplements.

Results from studies investigating the relationship between antioxidants from dietary supplements and MetS have been inconsistent. Czernichow et al. [13] reported that long-term supplementation of vitamin C, vitamin E, and β-carotenoid was not associated with the risk of MetS. Other intervention studies have also failed to show a beneficial effect of vitamin E and β-carotene supplementation on the risk factors of MetS [14,15,16]. However, some intervention studies have reported a beneficial effect of vitamin E supplementation on MetS risk factors [17,18].

The concept of total antioxidant capacity (TAC) was introduced because the evaluation of individual antioxidants does not reflect the antioxidant capacity of a whole diet or the interactions and synergistic effects of various antioxidants [19,20]. The association between TAC from diet and MetS has been reported in previous studies. Higher dietary TAC has been associated with low oxidative stress, low risk of abdominal obesity, and diabetes mellitus [21,22]. However, research examining the association between TAC from dietary supplements and the risk of MetS is scarce, not only in Korea, but also internationally. One study reported that higher intakes of antioxidant vitamins and higher serum total antioxidant status (TAS) from antioxidant supplementation were associated with a decreased waist circumference and low-density lipoprotein (LDL) to high-density lipoprotein (HDL) cholesterol ratio [23].

Therefore, this study sought to determine the status of antioxidant intake from dietary supplements and to examine the association between antioxidants and TAC from dietary supplements and the prevalence of MetS in Korean adults.

## 2. Methods

### 2.1. Study Subjects

The KNHANES is an annually conducted national survey to monitor and assess the health and nutritional status of Koreans and has a complex sampling design for selecting participants to represent the Korean population. The KNHANES consists of three parts, including a health interview, a health examination, and a nutrition survey that questions the use of dietary supplements. Dietary supplements are defined by the KNHANES as “a product taken for supplementing insufficient nutrients in the usual diet or for health promotion, which should contain vitamins, minerals, and functional ingredients in any form of tablets, capsules, powder, granules, and liquid”. Participants of the 2010 and 2011 KNHANES who were 19~64 years old included 10,230 adults; 8349 subjects completed all three components, including the health interview, health examination, and nutrition survey. To identify dietary supplement users, the response to “Have you been taking dietary supplements continuously for more than 2 weeks during the past year?” regarding dietary supplement consumption in the nutrition survey was used. Of the participants included in the study, 1938 subjects answered “yes” to the question and completed the supplement product names and supplement intake values (which can be identified in the 24-h recall data). In contrast, 4550 subjects answered “no” to the question. We excluded 1861 subjects who did not respond to the question (*n* = 3); who answered “yes” to the question, but did not take dietary supplements on the previous day per the 24-h recall interview, so their supplement intake values were not available (*n* = 1831); and who provided unidentified product names for the dietary supplement in the 24-h recall data (*n* = 27). We also excluded subjects whose daily energy intake was extremely high or low (<500 kcal/day or ≥5000 kcal/day) (*n* = 105), and whose antioxidant vitamin intakes from dietary supplements were extremely high (>99th percentile) (*n* = 75). Finally, a total of 6308 adults were confirmed as dietary supplement users (*n* = 1847) or as non-users (*n* = 4461). The KNHANES was approved by the Institutional Review Board of the Korea Centers for Disease Control and Prevention (2010-02CON-21-C, 2011-02CON-06-C).

### 2.2. Estimation of Antioxidant Intake from Diet and Dietary Supplements

The antioxidant intake from diet of individual subjects was estimated by linking the KNHANES 24-h recall data to an antioxidants and TAC database for common Korean foods developed by the Graduate School of Public Health at Seoul National University [24,25,26]. The antioxidants and TAC database consists of 3193 food items consumed by KNHANES subjects from the 2007~2012 survey. The database includes the contents of 43 individual antioxidants and the TAC per food item. The individual antioxidants included retinol, vitamin C, four forms of vitamin E (α-tocopherol, β-tocopherol, γ-tocopherol, and δ-tocopherol), six forms of carotenoids (α-carotene, β-carotene, β-cryptoxanthin, lutein, lycopene, and zeaxanthin), and seven flavonoid subclasses (flavonols, flavones, flavanones, flavan-3-ols, anthocyanins, isoflavones, and proanthocyanidins) with their 31 different components. A detailed description of the antioxidants and TAC database is available elsewhere [24,25,26]. The individual antioxidant intake of subjects was calculated by multiplying the amount of each food intake by the antioxidant content of each food consumed. In this study, vitamin A, retinol, carotenoids, vitamin C, vitamin E, and flavonoid intakes from diet were estimated via this method.

Vitamin A and vitamin C intakes from dietary supplements were estimated using the KNHANES 24-h recall data. Intake of other antioxidants, such as retinol, vitamin E, and β-carotene, from dietary supplements were estimated by the following processes. Dietary supplements have two categories: nutraceutical formulations and health functional foods. The components of dietary supplements in nutraceutical formulations were searched via the Korean Medical Library Engine (www.kmle.co.kr) and Druginfo (www.druginfo.co.kr), which are websites reporting nutraceutical formulations referenced in the KNHANES. We also investigated components of the products through the manufacturers’ websites or by telephone when the products were unavailable on the referenced websites. For health functional foods, we first searched the websites referenced by the KNHANES; if the referenced websites were not available, the health functional food search engine (http://www.foodsafetykorea.go.kr) of the Korean Ministry of Food and Drug Safety and manufacturers’ websites were used to find the products and components. In the case of foreign dietary supplements, ingredient information was collected from the Dietary Supplement Label Database of the National Institutes of Health. Finally, if the components were not found via these processes, a picture of the supplement’s label or a telephone call to the company were used to determine the ingredients.

Flavonoid and carotenoid intake (except for β-carotene) from dietary supplements could not be included in this study due to the lack of database. As a result, intakes of vitamin A, retinol, carotenoids (β-carotene), vitamin C, and vitamin E from dietary supplements were estimated in the study.

### 2.3. Estimation of TAC from Diet and Dietary Supplements

TAC values from diet and dietary supplements were estimated using the antioxidants and TAC database, which contains the antioxidant capacity of each food item [24,25,26]. TAC (mg vitamin C equivalents (VCE)) from an individual food item consumed was obtained by multiplying the amount of intake (g) by the antioxidant capacity (mg VCE/100 g) of the food item. Daily TAC (mg VCE) from diet was calculated via the sum of the TAC from all foods consumed by a subject from the one-day 24-h recall data. To estimate TAC from dietary supplements, TAC from individual antioxidants was calculated by multiplying the amount of intake of each antioxidant from the dietary supplements by the individual antioxidant capacity. Daily TAC from dietary supplements was estimated by summing the TAC from the supplements consumed by a subject.

Since vitamin E has different antioxidant capacities depending on the type of each isomer, the vitamin E components of dietary supplements consumed by subjects were determined to estimate TAC through the process mentioned above. Out of 926 dietary supplements containing vitamin E, there were 348 product types, excluding 578 duplicates. A sample of 70 product types was investigated, and the vitamin E components of 92.9% of the dietary supplements were identified as α-tocopherol. Therefore, TAC from vitamin E in dietary supplements was calculated by assuming that all vitamin E components taken by the subjects were α-tocopherol. The remaining 7.1% of the products provided their ingredient information as a mixture of vitamin E (*n* = 1), mixed tocopherol (*n* = 1), vitamin E only (*n* = 2), or tocopherol (*n* = 1), so that their specific components were unknown.

### 2.4. Diagnosis of MetS

Based on the health examination data of the KNHANES, the National Cholesterol Education Program Adult Treatment Panel III 2011 criteria were used to diagnose MetS [27]. Risk factors for MetS include high triglyceride levels (≥150 mg/dL), low HDL-cholesterol levels (men < 40 mg/dL, women < 50 mg/dL), high blood pressure (≥130/85 mmHg), high fasting blood glucose (≥100 mg/dL), and abdominal obesity (waist circumference of men ≥90 cm, women ≥85 cm for Koreans) [27,28]. MetS was diagnosed in the event that three or more risk factors were present in a subject.

### 2.5. Statistical Analysis

All Statistical analyses were performed using the SAS program (Statistical Analysis System, version 9.4, SAS Institute Inc., Cary, NC, USA). Clusters, strata, and survey weights were included for all analyses to adjust for the survey design of KNHANES, which enabled the results to be a representation of the Korean population. Categorical variables were tested with the Rao Scott Chi-square test using the PROC SURVEYFREQ procedure (Statistical Analysis System, version 9.4, SAS Institute Inc., Cary, NC, USA), and continuous variables were compared via Student’s *t* test. Antioxidant intakes are presented as means and standard errors (SEs).

Sociodemographic characteristics based on the health survey data of the KNHAES were used in the analysis as confounding variables. Educational levels were classified into four categories: ≤elementary school, middle school, high school, and ≥college. Household income levels were also divided into the lowest, lower middle, upper middle, and the highest. Alcohol consumption was defined as “yes” when a subject consumed alcohol more than once a month over the past one year. We defined current smoking as “yes” if a study subject had smoked more than 100 cigarettes in their lifetime and has continued to smoke. Regular physical activity was defined as “yes” if a study subject performed moderate physical activity more than 5 days a week for at least 30 min.

The odds ratio (OR) and 95% confidence intervals (CIs) of MetS and its risk factors according to dietary supplement use and antioxidant intake levels were estimated by multiple logistic regression analysis using the PROC SURVEYLOGSITC procedure, adjusted for age, sex, educational level, household income level, alcohol consumption, current smoking, and energy. All statistical significance levels were α = 0.05.

## 3. Results

### 3.1. General Characteristics of Study Subjects

Out of 6308 subjects, 1847 (24.5%) were classified as dietary supplement users and the others (75.5%) were classified as non-users (Table 1). The proportion of women (60.9%) who were dietary supplement users was higher than that of men (39.1%). Age, education, and household income levels of dietary supplement users were significantly higher than those of non-users (*p* < 0.05). However, there were no significant differences in the proportion of subjects with obesity, regular physical activity, and macronutrient intakes between dietary supplement users and non-users. Dietary supplement users were also less likely to drink alcohol and smoke cigarettes (*p* < 0.05).

### 3.2. Antioxidant Intakes and Contribution of Dietary Supplements

The status of antioxidant intake of dietary supplement users and non-users is shown in Table 2. There were no significant differences in antioxidant intakes from diet between dietary supplement users and non-users, except for vitamin A and vitamin E. However, when comparing total antioxidant intake between the two groups, including dietary supplements, the intake of vitamin A, retinol, vitamin C, vitamin E, and TAC in dietary supplement users was greater than that in non-users (*p* < 0.05). When adjusting for energy intake, the antioxidant intake density (each unit/1000 kcal/day) from diet was not different between the two groups, except for vitamin E intake. However, the intake of vitamin A, retinol, vitamin C, vitamin E, and TAC of dietary supplement users was significantly greater than that in non-users when including dietary supplements (*p* < 0.05).

Table 2 also shows the contribution (%) of dietary supplements to total antioxidant intake. After adjusting for energy intake, vitamin C was the most contributing antioxidant (68.8%) from dietary supplements, followed by vitamin E (41.8%), retinol (21.1%), and vitamin A (20.9%). The average contribution of dietary supplements for antioxidant intakes was 30.8%. Furthermore, the average TAC from supplements in dietary supplement users was 140.6 mg VCE, contributing to 38% of total TAC.

### 3.3. Association Between Antioxidant Intake and MetS

Table 3 shows the association between antioxidant intake and MetS. The prevalence of MetS was significantly lower in dietary supplement users than that in non-users (OR = 0.82; 95% CI, 0.68–0.98). Among dietary supplement users, the highest tertile for vitamin A (OR = 0.72; 95% CI, 0.53–0.99) and E (OR = 0.74; 95% CI, 0.55–0.99) intake showed a lower prevalence for MetS than those in the lowest tertile among non-users. Subjects in the highest tertile of TAC among dietary supplement users showed a lower prevalence of Mets (OR = 0.72; 95% CI, 0.52–0.99) than that in non-users. Antioxidant intake from diet or supplements was not associated with elevated triglycerides, elevated blood pressure, or elevated fasting glucose.

## 4. Discussion

In this study using KNHANES (2010~2011) data, we found that intakes of vitamin A, vitamin E, and TAC from dietary supplements are inversely associated with MetS among Korean adults.

The epidemiological characteristics of dietary supplement users, such as more women, higher education, and higher household income, suggest dietary supplement users were more likely to have a healthy lifestyle (e.g., non-drinking and non-smoking) and were comparable with previous reports conducted by Kang et al. [11], Radimer et al. [29], and Kofoed et al. [30]. The use of dietary supplements is known to be associated with an individual’s self-consciousness toward having a healthy diet [31], so that the greater the desire to have a healthy diet, the greater the likelihood that the individual will use dietary supplements [31].

When we examined antioxidant intake from dietary supplements, vitamins C and E were the most contributing antioxidants to total antioxidant intake (68.8% and 41.8%, respectively). Furthermore, 38% of TAC was supplied by dietary supplements, and TAC was 2 times higher in dietary supplement users than in non-users. Chun et al. [12,20] reported that dietary supplements are the main sources of vitamin C, vitamin E, and TAC, contributing 54%, 64%, and 25% of total intake, respectively, in US adults. Considering that vitamin and mineral supplements were the most frequently consumed supplements among Koreans [11], it was not surprising that dietary supplements were the major source of vitamin C, vitamin E, and TAC in this study.

Regarding the association between antioxidant intake and MetS, we found the prevalence of MetS to be significantly lower in dietary supplement users than that in non-users (OR = 0.82; 95% CI, 0.68–0.98). Previous studies have reported that MetS patients have lower blood concentrations of vitamin A, vitamin C, and carotenoids [32,33,34], and elevated levels of oxidative stress and inflammation [35,36,37], although clear mechanisms have not been elucidated. Supplementation with antioxidant vitamins has been shown to increase the blood concentration of vitamins and decrease oxidative stress in several intervention studies [38,39]. Hercberg et al. [38] reported an increase in the concentration of serum antioxidants after supplementing with 120 mg vitamin C, 30 mg vitamin E, 6 mg β-carotene, 100 μg selenium, and 20 mg zinc every day for 7 years. Actis-Goretta et al. [39] also reported that vitamin E supplementation decreases oxidative stress by 11%. Thus, the intake of antioxidants from dietary supplements may have beneficial effects on MetS by increasing blood concentrations, which may reduce oxidative stress in the body.

Among antioxidant vitamins consumed from dietary supplements, vitamin A showed beneficial effects on abdominal obesity in this study. Vitamin A and its metabolites are known to be important regulators of obesity [40]. Retinoic acid, a vitamin A metabolite, is involved in adipocyte and preadipocyte apoptosis, thereby inhibiting fat production [41]. Previous studies reported an inverse association between serum vitamin A levels and obesity [42,43]. In addition, Zulet et al. [44] reported that vitamin A intake is inversely related to adiposity, with lower weights, lower body mass indexes (BMIs), and lower waist circumferences among those with higher vitamin A intakes. Thus, an adequate intake of vitamin A from diet and dietary supplements may help reduce the risk of MetS. The high vitamin A intake in dietary supplement users was directly related to HDL-cholesterol in this study. Previous studies have shown that this association differs for carotenoids and retinol. In the case of retinol, an intervention study by Farhangi et al. [45] reported a decrease in HDL-cholesterol levels in a group that supplemented with retinyl palmitate, while an increase in serum HDL-cholesterol levels was shown in the group receiving β-carotene [46,47]. Mooradian et al. [48] also reported that the effect of increasing or decreasing serum HDL-cholesterol differs depending on the type of dietary supplement. Therefore, to clarify the relationship between vitamin A intake via dietary supplements and HDL-cholesterol levels, further studies are needed to elucidate the effects of vitamin A supplement types on MetS.

Vitamin E intake in dietary supplement users was inversely related to MetS and abdominal obesity in this study. Vitamin E is a powerful chain-breaking antioxidant that reduces the level of oxidative stress and prevents oxidative damage, which play important roles in the development of MetS [49]. Reductions in oxidative stress associated with vitamin E supplementation have been reported in both healthy adults and patients with MetS [17,50]. Wang et al. [17] compared the levels of oxidative stress and blood lipids of women with MetS after having taken α-tocopherol or placebo for 4 months and found that oxidative stress and total cholesterol decreased in the group of α-tocopherol [17]. Thus, vitamin E intake from dietary supplements may have a beneficial effect on MetS by reducing oxidative stress related to the development of MetS. Several cross-sectional studies have suggested that serum tocopherol concentrations are negatively associated with obesity [43,51]; however, there are contradictory studies that report a positive correlation between serum α-tocopherol levels and abdominal obesity [52]. In addition, there was a significant difference in vitamin E intake from diet between dietary supplement users and non-users in this study; therefore, it is difficult to determine whether the low prevalence of MetS in dietary supplement users were attributable to the dietary supplements.

High TAC from dietary supplements was also inversely associated with MetS in this study. Previous studies have reported a protective role of dietary TAC against oxidative stress and inflammation, which are involved in the development of MetS [21,53,54]. An inverse association between dietary TAC and risk of cerebrovascular disease related to oxidative stress and inflammation [55] was found in an Italian cohort of the European Prospective Investigation into Cancer and Nutrition (EPIC) study. Regarding dietary supplements, Kim et al. [23] reported dietary supplements significantly increase total antioxidant status, an indicator of TAC in plasma, and decrease waist circumference and the LDL/HDL cholesterol ratio, which are related to the risk factors of MetS. Thus, an adequate antioxidant intake from dietary supplements, ensuring sufficient TAC, may have a positive effect on MetS. However, we cannot exclude the possibility of high TAC levels from the diet of dietary supplement users, even though there were no statistically significant differences in TAC from diet between dietary supplement users and non-users. One factor that could affect TAC is antioxidant bioavailability in vivo because TAC is a parameter that is estimated in vitro [55]. Antioxidant bioavailability differs depending on the type of antioxidant, such as methylated flavonoids [56], cooking and processing conditions [57,58], and digestion stages, including the influence of microflora in the colon [58]. Thus, well designed studies to determine the relationship between TAC and MetS that also consider antioxidant bioavailability are required.

There are some limitations to this study. First, flavonoids and other carotenoids, except for β-carotene, were not included in the estimates for antioxidant intake from dietary supplements, since the KNHANES did not supply those data. Thus, antioxidant intake and TAC might be underestimated. Second, the vitamin E component of supplements was assumed to be α-tocopherol via sample examination and used to estimate the TAC of dietary supplement users; therefore, measurement errors for estimating TAC might be increased in dietary supplement users in this study. Third, we found differences in several general characteristics such as sex, age, education level, alcohol consumption, and current smoking status between identified dietary supplement users and dietary supplement users who did not take the supplements on the previous day, as reported in the 24-h recall interview, and were excluded from this study; thus, further study including those dietary supplement users will be needed. Further, we could not rule out the possibility that antioxidants differ in bioavailability between foods that are cooked or processed, which could affect TAC of the food. Finally, as a cross-sectional study, it is unclear whether the intake of antioxidants and TAC from supplements caused a reduction in MetS risk. Despite these limitations, this study has strengths in terms of estimating the antioxidant intake from diet and supplements in Koreans and supplying information regarding the association between antioxidant intake from supplements and MetS based on a large sample of Korean adults.

## 5. Conclusions

In conclusion, 30.8% of antioxidants and 38% of TAC were supplied on average by dietary supplements, suggesting that dietary supplements are quite important sources of antioxidant intake and TAC among Korean adults. Dietary supplement users had a lower prevalence of MetS than non-users. Intakes of vitamin A, vitamin E, and TAC from supplements were associated with a decreased prevalence of MetS and its component factors, including abdominal obesity and reduced HDL-cholesterol. A more comprehensive investigation of dietary supplements to clarify the effects on MetS is necessary.

## Figures and Tables

**Table 1:** General characteristics of non-users and dietary supplement users among Korean adults.

Characteristics	Total *n* (%)	Non-Users *n* (%)	DS Users *n* (%)	*p*-Value ^1^
**Total**	6308 (100)	4461 (75.5)	1847 (24.5)	
**Sex**				<0.0001
Male	2599 (52.6)	2044 (57.0)	555 (39.1)	
Female	3709 (47.4)	2417 (43.0)	1292 (60.9)	
**Age**				<0.0001
19–29	931 (23.0)	776 (26.0)	155 (13.7)	
30–49	2947 (48.7)	2137 (48.7)	810 (48.6)	
50–64	2430 (28.3)	1548 (25.3)	882 (37.7)	
**Education**				0.0025
≤Elementary	892 (11.2)	636 (11.2)	256 (11.1)	
Middle school	715 (10.3)	486 (10.1)	229 (11.1)	
High school	2424 (43.2)	1766 (44.6)	658 (38.8)	
≥College	2171 (35.3)	1493 (34.1)	678 (39.0)	
**Household income** ^2^				<0.0001
Lowest	680 (11.3)	535 (12.3)	147 (8.3)	
Lower middle	1639 (28.0)	1219 (28.9)	420 (25.5)	
Upper middle	2017 (32.8)	1423 (33.0)	594 (32.0)	
Highest	1905 (27.9)	1233 (25.8)	672 (34.2)	
**BMI**				NS
<23	2915 (45.5)	2022 (44.6)	893 (48.2)	
23–25	1429 (22.8)	997 (22.8)	432 (22.6)	
≥25	1964 (31.7)	1442 (32.5)	522 (29.2)	
**Alcohol consumption** (Yes) ^3^	3514 (61.7)	2620 (64.5)	894 (52.9)	<0.0001
**Current smoking** (Yes) ^4^	1319 (27.6)	1089 (31.2)	230 (16.5)	<0.0001
**Regular physical activity** (Yes) ^5^	615 (10.1)	436 (10.1)	179 (10.1)	NS
**Nutrient intakes (mean ± SE)**				NS
Energy (kcal/day)	2124.0 ± 15.0	2135.8 ± 18.1	2088.9 ± 25.4	
Carbohydrate (g/day)	332.3 ± 2.4	331.2 ± 2.7	336.0 ± 4.1	
Protein (g/day)	77.6 ± 0.7	77.5 ± 0.8	77.9 ± 1.2	
Fat (g/day)	45.6 ± 0.5	46.1 ± 0.7	44.2 ± 0.9	

* All values are presented as *n* (%) except for the nutrient intakes; ^1^ *T*-test was used for continuous variables and chi-square test was used for categorical variables with appropriate cluster, strata, and weight; ^2^ The lowest means first quartile, lower middle means second quartile, upper middle means third quartile, and the highest means forth quartile; ^3^ “Yes” means subject consumed alcohol more than once a month over the past year; ^4^ “Yes” means subject smoked at least 100 cigarettes in entire life and currently smokes cigarettes; ^5^ “Yes” means subject participates in moderate physical activity more than 5 days per week for at least 30 min. DS, dietary supplement; BMI, body mass index; S.E, standard error.

**Table 2:** Antioxidant intakes from diet and supplements by the status of dietary supplement consumption among Korean adults.

Antioxidants	Non-Users (*n* = 4461)	DS Users (*n* = 1847)				*p*-Value ^2^	*p*-Value ^3^
	Diet	Diet	DS	Total ^1^	Supplement Contribution (%)		
Antioxidant intake (each unit/day)							
Vitamin A (µgRE)	800.4 ± 18.2	902.0 ± 31.0	203.2 ± 14.4	1105.1 ± 35.5	18.4	0.0133	<0.0001
Retinol (µg)	126.0 ± 8.8	143.6 ± 13.4	30.7 ± 5.5	174.3 ± 14.3	17.6	0.1065	0.0009
Carotenoids (µg)	9033.6 ± 248.7	9955.4 ± 341.8	113.9 ± 17.2	10,069.0 ± 341.8	1.1	0.1452	0.0789
Vitamin C (mg)	104.7 ± 3.3	126.4 ± 4.5	252.1 ± 14.9	378.5 ± 15.2	66.6	0.0522	<0.0001
Vitamin E (mg)	15.0 ± 0.2	16.1 ± 0.3	10.0 ± 0.9	26.2 ± 1.0	38.2	<0.0001	<0.0001
Flavonoids (mg)	198.5 ± 7.7	241.5 ± 9.9	-	241.5 ± 9.9	-	0.0506	-
TAC (mg VCE)	378.0 ± 13.4	459.7 ± 17.7	254.9 ± 14.9	714.5 ± 22.5	35.7	0.0601	<0.0001
Antioxidant density (each unit/1000 kcal/day)							
Vitamin A (µgRE)	385.1 ± 7.8	433.2 ± 12.6	114.7 ± 8.1	547.9 ± 15.9	20.9	0.2773	<0.0001
Retinol (µg)	56.8 ± 3.2	65.4 ± 5.1	17.5 ± 3.2	82.9 ± 6.0	21.1	0.1406	0.0002
Carotenoids (µg)	4426.6 ± 115.7	4926.5 ± 167.4	67.7 ± 11.7	4994.3 ± 167.3	1.4	0.6027	0.3695
Vitamin C (mg)	51.0 ± 1.5	63.0 ± 2.1	139.0 ± 8.1	202.0 ± 8.1	68.8	0.0997	<0.0001
Vitamin E (mg)	7.1 ± 0.1	7.8 ± 0.1	5.6 ± 0.5	13.4 ± 0.5	41.8	0.0001	<0.0001
Flavonoids (mg)	96.7 ± 3.3	121.8 ± 4.9	-	121.8 ± 4.9	-	0.0826	-
TAC (mg VCE)	183.8 ± 6.1	229.8 ± 8.6	140.6 ± 8.1	370.4 ± 11.0	38.0	0.1516	<0.0001

* All values are presented as mean ± SE, except for supplement contribution (%); ^1^ sum of antioxidant intakes from diet and dietary supplements of users; ^2^ *p*-values for the difference in antioxidant intake from diet between non-users and users adjusted for age, sex, education level, household income, alcohol consumption, and current smoking; ^3^ *p*-values for the difference in total antioxidant intake between non-users and users adjusted for age, sex, education level, household income, alcohol consumption, and current smoking. DS, dietary supplement; VCE, vitamin C equivalents; TAC, total antioxidant capacity; RE, retinol equivalents; S.E, standard error.

**Table 3:** Association between total antioxidant intake and MetS prevalence among Korean adults.

MetS and Its Risk Factors	Non-Users (*n* = 4461)			DS Users (*n* = 1847)			Total (*n* = 6308)		
MetS OR (95%CI) ^1^	1			**0.82 (0.68–0.98)**					
	Antioxidant intake level ^2^						Antioxidant intake level ^2^		
	T1	T2	T3	T1	T2	T3	T1	T2	T3
Metabolic syndrome									
Vitamin A	1	1.10 (0.86–1.41) ^3^	0.93 (0.74–1.17)	0.79 (0.59–1.06)	0.97 (0.72–1.31)	0.72 (0.53–0.99)	1	1.03 (0.83–1.27)	0.90 (0.73–1.10)
Retinol	1	1.17 (0.90–1.52)	1.17 (0.93–1.47)	0.97 (0.73–1.30)	1.01 (0.73–1.38)	0.73 (0.52–1.03)	1	1.08 (0.88–1.34)	1.05 (0.87–1.28)
Carotenoids	1	1.10 (0.87–1.39)	0.96 (0.76–1.23)	0.82 (0.61–1.10)	0.81 (0.59–1.10)	0.89 (0.66–1.18)	1	0.96 (0.79–1.16)	0.96 (0.79–1.16)
Vitamin C	1	1.07 (0.85–1.34)	1.04 (0.82–1.32)	0.94 (0.68–1.28)	0.84 (0.62–1.13)	0.75 (0.56–1.02)	1	1.07 (0.88–1.29)	0.84 (0.68–1.04)
Vitamin E	1	0.91 (0.71–1.15)	0.98 (0.77–1.23)	0.79 (0.58–1.08)	0.82 (0.60–1.14)	0.74 (0.55–0.99)	1	0.97 (0.80–1.19)	0.95 (0.78–1.16)
Flavonoids	1	0.97 (0.77–1.23)	0.99 (0.79–1.24)	-	-	-	1	1.06 (0.87–1.29)	0.95 (0.79–1.15)
TAC	1	1.01 (0.79–1.28)	0.94 (0.73–1.20)	0.93 (0.69–1.25)	0.73 (0.54–0.99)	0.72 (0.52–0.99)	1	1.06 (0.87–1.28)	0.86 (0.71–1.05)
Abdominal obesity ^4^									
Vitamin A	1	1.07 (0.87–1.32)	1.19 (0.96–1.47)	0.88 (0.68–1.15)	0.94 (0.70–1.26)	0.83 (0.63–1.11)	1	1.09 (0.91–1.30)	1.07 (0.89–1.28)
Retinol	1	1.08 (0.86–1.35)	1.04 (0.84–1.29)	0.87 (0.66–1.14)	0.73 (0.56–0.96)	0.94 (0.71–1.26)	1	0.99 (0.82–1.20)	1.06 (0.88–1.28)
Carotenoids	1	1.24 (1.01–1.53)	1.10 (0.89–1.36)	0.89 (0.69–1.17)	0.89 (0.67–1.18)	0.94 (0.72–1.24)	1	1.13 (0.95–1.34)	1.07 (0.90–1.27)
Vitamin C	1	0.92 (0.74–1.14)	0.97 (0.78–1.20)	0.81 (0.63–1.05)	0.76 (0.57–1.02)	0.77 (0.58–1.03)	1	0.94 (0.80–1.12)	0.87 (0.72–1.05)
Vitamin E	1	0.82 (0.66–1.02)	0.85 (0.69–1.05)	0.80 (0.60–1.06)	0.72 (0.53–0.96)	0.66 (0.52–0.84)	1	0.86 (0.72–1.04)	0.82 (0.69–0.97)
Flavonoids	1	0.84 (0.67–1.06)	0.90 (0.72–1.12)	-	-	-	1	0.89 (0.74–1.06)	0.93 (0.77–1.12)
TAC	1	0.96 (0.77–1.20)	0.99 (0.80–1.23)	0.85 (0.66–1.10)	0.75 (0.56–1.00)	0.79 (0.59–1.04)	1	1.01 (0.84–1.21)	0.92 (0.76–1.12)
Elevated triglycerides ^5^									
Vitamin A	1	0.96 (0.78–1.19)	0.84 (0.69–1.02)	0.81 (0.59–1.11)	0.89 (0.69–1.16)	0.78 (0.58–1.04)	1	1.03 (0.87–1.23)	0.87 (0.73–1.02)
Retinol	1	1.04 (0.83–1.30)	0.98 (0.79–1.21)	0.90 (0.67–1.20)	1.04 (0.77–1.40)	0.75 (0.54–1.02)	1	1.01 (0.84–1.22)	0.95 (0.79–1.14)
Carotenoids	1	1.01 (0.84–1.23)	0.85 (0.69–1.05)	0.82 (0.60–1.11)	0.83 (0.63–1.08)	0.91 (0.69–1.19)	1	0.93 (0.79–1.11)	0.91 (0.76–1.08)
Vitamin C	1	1.13 (0.92–1.39)	1.09 (0.88–1.37)	1.01 (0.75–1.36)	0.98 (0.74–1.29)	0.87 (0.63–1.21)	1	1.17 (0.98–1.40)	0.95 (0.78–1.15)
Vitamin E	1	1.05 (0.85–1.29)	1.00 (0.80–1.24)	0.95 (0.70–1.28)	0.98 (0.74–1.30)	0.78 (0.59–1.03)	1	0.95 (0.79–1.14)	0.93 (0.78–1.11)
Flavonoids	1	0.89 (0.72–1.10)	0.90 (0.72–1.12)	-	-	-	1	0.96 (0.80–1.15)	0.90 (0.75–1.09)
TAC	1	1.02 (0.83–1.25)	0.96 (0.78–1.19)	0.99 (0.75–1.32)	0.79 (0.59–1.06)	0.84 (0.61–1.15)	1	1.03 (0.86–1.24)	0.93 (0.76–1.13)
Reduced HDL-C ^6^									
Vitamin A	1	0.96 (0.81–1.16)	0.91 (0.75–1.10)	1.01 (0.79–1.29)	0.92 (0.72–1.17)	0.72 (0.57–0.93)	1	0.88 (0.75–1.03)	0.88 (0.75–1.03)
Retinol	1	1.13 (0.93–1.38)	1.03 (0.85–1.24)	1.05 (0.82–1.35)	0.97 (0.76–1.23)	0.90 (0.69–1.18)	1	1.09 (0.92–1.28)	1.01 (0.86–1.18)
Carotenoids	1	0.96 (0.80–1.17)	1.13 (0.94–1.36)	0.98 (0.78–1.25)	0.94 (0.74–1.20)	0.92 (0.73–1.17)	1	0.95 (0.80–1.12)	1.08 (0.92–1.28)
Vitamin C	1	1.29 (1.06–1.58)	1.20 (0.99–1.46)	1.13 (0.88–1.45)	1.12 (0.87–1.44)	0.98 (0.76–1.26)	1	1.16 (0.99–1.36)	1.06 (0.89–1.25)
Vitamin E	1	1.04 (0.86–1.25)	1.15 (0.95–1.4)	0.92 (0.71–1.2)	1.22 (0.96–1.56)	0.83 (0.66–1.05)	1	1.07 (0.91–1.27)	1.07 (0.91–1.26)
Flavonoids	1	1.00 (0.82–1.21)	1.17 (0.96–1.43)	-	-	-	1	1.06 (0.90–1.24)	1.15 (0.97–1.35)
TAC	1	0.92 (0.76–1.11)	1.19 (0.97–1.45)	0.88 (0.68–1.13)	1.14 (0.89–1.45)	0.87 (0.67–1.13)	1	1.09 (0.93–1.28)	1.13 (0.96–1.33)
Elevated blood pressure ^7^									
Vitamin A	1	1.05 (0.84–1.32)	1.08 (0.86–1.35)	1.30 (0.94–1.81)	1.01 (0.74–1.39)	0.85 (0.62–1.15)	1	1.03 (0.84–1.25)	1.00 (0.82–1.21)
Retinol	1	0.98 (0.79–1.22)	1.03 (0.82–1.29)	1.26 (0.94–1.68)	0.94 (0.69–1.27)	0.86 (0.63–1.17)	1	0.89 (0.73–1.07)	0.89 (0.73–1.10)
Carotenoids	1	1.12 (0.89–1.40)	1.00 (0.8–1.26)	1.22 (0.87–1.71)	1.07 (0.78–1.48)	0.85 (0.62–1.17)	1	1.02 (0.84–1.24)	0.93 (0.77–1.13)
Vitamin C	1	1.04 (0.83–1.30)	0.92 (0.72–1.18)	1.09 (0.79–1.50)	0.91 (0.65–1.26)	0.96 (0.67–1.39)	1	1.04 (0.85–1.27)	0.84 (0.69–1.03)
Vitamin E	1	0.98 (0.78–1.22)	0.87 (0.68–1.11)	1.02 (0.75–1.40)	0.92 (0.67–1.27)	0.93 (0.69–1.26)	1	0.94 (0.77–1.14)	0.94 (0.77–1.16)
Flavonoids	1	0.96 (0.75–1.22)	0.96 (0.76–1.22)	-	-	-	1	0.99 (0.82–1.19)	0.98 (0.80–1.19)
TAC	1	1.07 (0.85–1.34)	0.93 (0.74–1.18)	1.04 (0.77–1.40)	1.03 (0.76–1.40)	0.94 (0.67–1.31)	1	1.05 (0.88–1.26)	0.91 (0.74–1.12)
Elevated fasting glucose ^8^									
Vitamin A	1	1.05 (0.83–1.32)	1.01 (0.81–1.26)	0.79 (0.59–1.07)	1.02 (0.76–1.38)	1.11 (0.83–1.48)	1	1.09 (0.90–1.32)	1.17 (0.96–1.42)
Retinol	1	0.99 (0.80–1.22)	0.92 (0.72–1.17)	0.85 (0.63–1.14)	1.01 (0.76–1.36)	0.91 (0.67–1.23)	1	1.01 (0.84–1.22)	0.93 (0.76–1.14)
Carotenoids	1	1.13 (0.89–1.43)	1.12 (0.88–1.43)	0.94 (0.70–1.27)	1.01 (0.74–1.38)	1.14 (0.84–1.55)	1	1.03 (0.85–1.25)	1.13 (0.92–1.39)
Vitamin C	1	0.94 (0.74–1.18)	0.94 (0.73–1.22)	0.86 (0.63–1.17)	0.95 (0.71–1.27)	0.93 (0.68–1.27)	1	0.93 (0.76–1.13)	0.96 (0.78–1.18)
Vitamin E	1	0.98 (0.78–1.24)	1.02 (0.8–1.28)	0.89 (0.66–1.21)	0.85 (0.63–1.15)	0.99 (0.74–1.34)	1	0.98 (0.81–1.18)	1.08 (0.89–1.31)
Flavonoids	1	1.04 (0.82–1.32)	1.00 (0.80–1.27)	-	-	-	1	1.16 (0.95–1.40)	1.00 (0.81–1.22)
TAC	1	1.04 (0.82–1.31)	0.86 (0.68–1.10)	0.91 (0.67–1.25)	0.85 (0.62–1.16)	0.97 (0.71–1.33)	1	1.05 (0.85–1.30)	0.89 (0.73–1.10)

* All values are presented as odds ratio (95% confidence interval); ^1^ odds ratio was adjusted for age, sex, education level, household income, alcohol consumption, current smoking, and energy; ^2^ unit for antioxidant: vitamin A (µg retinol equivalents (RE)/1000 kcal/day), retinol (µg/1000 kcal/day), carotenoids (µg/1000 kcal/day), vitamin C (mg/1000 kcal/day), vitamin E (mg/1000 kcal/day), flavonoids (mg/1000 kcal/day), and total antioxidant capacity (TAC, mg vitamin C equivalents (VCE)/1000 kcal/d); ^3^ odds ratio was adjusted for age, sex, education level, household income, alcohol consumption, and current smoking; ^4^ abdominal obesity: waist circumference of men ≥ 90 cm, women ≥ 85 cm for Koreans; ^5^ elevated triglycerides: ≥150 mg/dL; ^6^ reduced HDL-C: men < 40 mg/dL, women < 50 mg/dL; ^7^ elevated blood pressure: ≥130/85 mmHg; ^8^ elevated fasting glucose: ≥100 mg/dL. DS, dietary supplement; MetS, metabolic syndrome; HDL-C, high-density lipoprotein cholesterol; OR, odds ratio.
